# Modulation of Moisturizing and Barrier Related Molecular Markers by Extracellular Vesicles Derived from *Leuconostoc mesenteroides* DB-21 Isolated from *Camellia japonica* Flower

**DOI:** 10.3390/cimb47121022

**Published:** 2025-12-08

**Authors:** Junseok Baek, Seongguk Cho, Gibok Lee, Hosam Ki, Su Young Kim, Gyu-min Choi, Jae Hong Kim, Ji-Woong Kim, Chang-Min Park, Seung-Young Kim, Byeong-Min Choi, Yang Gyu Choi

**Affiliations:** 1 Materials Science Research Institute, LABIO Co., Ltd., Seoul 08501, Republic of Korea; 2 R&D Center, Hankook Cosmetics Manufacturing Co., Ltd., 35 Cheonggyecheon-ro, Jongno-gu, Seoul 03188, Republic of Korea; 3 Department of Pharmaceutical Engineering & Biotechnology, Sunmoon University, Asan-si 31460, Chungnam, Republic of Korea

**Keywords:** skin barrier, moisturizing, *Camellia japonica*, *Leuconostoc mesenteroides* DB-21, extracellular vesicles

## Abstract

Among the microorganisms present in the microbiome of *Camellia japonica* flowers, extracellular vesicles (EVs) derived from *Leuconostoc mesenteroides* were isolated to investigate their modulatory effects on moisturizing and barrier-related molecular markers. To identify the function of major proteins in *L. mesenteroides* DB-21-derived extracellular vesicles (LEVs), Gene Ontology (GO) analysis was performed, revealing ATP binding, ribosomal structural proteins, and metal ion binding as predominant molecular-function categories. These proteomic characteristics provide a molecular context that supports the interpretation of the moisturizing and barrier-related responses observed in this study. To further verify new findings, we performed functional evaluations using in vitro and 3D skin models. LEVs increased the mRNA expression level of HAS3, which encodes hyaluronic acid synthase. In addition, the expression levels of filaggrin and involucrin, key proteins involved in skin barrier formation, increased, and these markers were determined a concentration-dependent increase in a 3D artificial skin model. Also, we confirmed that the expression levels of filaggrin and involucrin, which were reduced by UVB damage, were restored when LEVs were applied. In conclusion, LEVs are effective in enhancing various molecular markers related to the skin barrier function, and these results reveal that they hold promise as next-generation microbiome-based functional ingredients.

## 1. Introduction

Extracellular vesicles (EVs) are generally known as lipid bilayer vesicles, ranging in size from 30 to 200 nm, and containing various biomolecules, including proteins, lipids, and nucleic acids. They reflect the state of the cell and play a crucial role in intercellular communication [1,2]. Early exosomes were proposed as microvesicles with 5′-nucleotidase activity [3]. Subsequent research identified exosomes for the first time during the process of transferrin receptor removal in reticulocytes [4,5,6].

The EV of bacteria was initially observed in *Escherichia coli* during the 1960s, where a glycolipid complex secreted extracellularly under lysine-limited conditions was identified [7]. Subsequently, a vesicle structure derived from the cell surface was elucidated [8]. In Gram-negative bacteria, these are designated as outer membrane vesicles (OMVs), which originate from the outer membrane, and have been extensively studied in relation to pathogenicity, survival, and host–microbe interactions [9,10]. Conversely, in Gram-positive bacteria, which were previously thought to have limited capacity to produce EVs due to their thick peptidoglycan layer, it was reported in 2009 that *Staphylococcus aureus* produces EVs that are comparable in size and protein composition to OMVs of Gram-negative bacteria [11,12]. These findings demonstrate that EV formation is a universal phenomenon intrinsic to both eukaryotic and prokaryotic cells.

Serving as the largest organ in the human body, the skin functions as a protective barrier against harmful substances and microorganisms while preventing water loss [13]. The epidermis is made up of four distinct layers known as the stratum basale, stratum spinosum, stratum granulosum, and stratum corneum [14]. In the epidermis, proteins such as involucrin (INV), filaggrin (FLG), and loricrin (LOR) are formed, along with lipids and natural moisturizing factors (NMF), during keratinocyte differentiation. These components maintain the skin barrier and help the skin stay balanced [15]. When skin is exposed to ultraviolet (UV) light, matrix metalloproteinases (MMPs) become more active. These enzymes break down collagen in the extracellular matrix, which weakens the dermis and barrier [16,17]. UV also increases reactive oxygen species (ROS), which leads to inflammatory responses and may accelerate skin aging. Prolonged UV exposure activates the p53 signaling pathway, inducing apoptosis, and results in the formation of damaged keratinocytes referred to as sunburn cells [18].

Various natural substances and microbial-derived EVs have recently been documented as contributors to skin-barrier reinforcement. Specifically, *Camellia japonica* flower extract was described as inhibiting UVA-induced MMP-1 expression and restoring type I procollagen, actions associated with anti-photoaging activity [19]. Evidence has also been presented for its ability to suppress melanin synthesis [20], alongside antioxidant and anti-inflammatory properties [21]. In atopic dermatitis-like animal models, symptoms were alleviated through the suppression of Immunoglobulin E (IgE) and histamine, reduction in oxidative stress, and restoration of the skin barrier [22]. Additionally, EVs and culture supernatant derived from probiotics have been reported to contribute to skin health and barrier function. *Lactiplantibacillus plantarum* LRCC5195 and its supernatant were shown to modulate immune responses in atopic dermatitis models by downregulating Th2 cytokines and enhancing IL-10 production, thereby supporting immune balance and indirectly helping to maintain the skin barrier [23]. EVs derived from *Bifidobacterium longum* from infants have been reported to strengthen the skin barrier by increasing the expression of FLG and LOR [24]. These findings support the biological activity of *Camellia japonica* flower extract and suggest that microorganisms obtained from its flowers may offer practical applications for improving barrier function.

Proteins in EVs are reported to be closely associated with the various biological responses mediated by EVs [25]. In particular, research results have been reported that the proteins in microbe-derived EVs can affect various processes relevant to skin homeostasis, including keratinocyte differentiation, modulation of inflammation, and the expression of barrier-related proteins [26,27,28]. Considering these prior studies, elucidating the proteins of microbial-derived EVs can serve as an essential initial step for understanding their potential functional characteristics and provide a foundational molecular basis for exploring skin-related functions.

*Leuconostoc mesenteroides* is a lactic acid bacterium found in kimchi, dairy products, and several plants [29,30,31]. It is also listed as GRAS (Generally Recognized as Safe) and may be considered a microorganism with proven safety. It has been studied in relation to whitening effects and anti-inflammatory activity [32]. Reports have shown that EVs from probiotics can increase the expression of FLG and fibronectin while lowering collagen degradation, which helps protect the skin from UV damage [33]. This study aimed to examine changes in the expression of skin barrier factors using extracellular vesicles (LEVs) from *Leuconostoc mesenteroides* DB-21, which were isolated and characterized from *Camellia japonica* flower. The goal was to assess their potential as cosmetic actives for supporting barrier integrity under environmental stress.

## 2. Materials and Methods

### 2.1. Incubation of Leuconostoc mesenteroides DB-21

The strain *Leuconostoc mesenteroides* DB-21 (KCTC 15909BP), isolated from native *Camellia japonica* flower in Korea, was supplied by Sunmoon University and utilized for research purposes. *L. mesenteroides* DB-21 was cultivated aerobically at a temperature of 30 °C using culture medium as de Man, Rogosa and Sharpe (MRS).

### 2.2. Isolation of LEVs

*L. mesenteroides* DB-21 was cultured in a modified MRS (mMRS) medium, with all animal-derived components substituted with plant-based alternatives, adhering to the standard MRS formulation. The culture was incubated at 30 °C for 18 h. Subsequently, cells were removed via centrifugation at 3500× *g* for 20 min at 4 °C. The resulting supernatant was then filtered through a 0.22 μm polyethersulfone (PES) membrane filter (Millipore, Burlington, MA, USA). The filtered culture supernatant was concentrated utilizing tangential flow filtration (TFF; Sartorius, Göttingen, Germany) with a regenerated cellulose cartridge possessing a 100 kDa nominal molecular weight cut-off (NMWC) [34]. During the TFF process, the transmembrane pressure was maintained below 0.5 bar, and after the culture supernatant was concentrated approximately 20-fold, it was washed twice with sterile PBS (Welgene, Gyeongsan, Republic of Korea). The final EVs obtained were filtered through a 0.22 μm polyethersulfone (PES) membrane filter (Millipore, Burlington, MA, USA) and stored at 4 °C.

### 2.3. Characterization and Morphology of LEVs

LEVs were analyzed employing nanoparticle tracking analysis (NTA) and transmission electron microscopy (TEM). For NTA, the samples were diluted with sterile-filtered PBS to achieve an appropriate concentration (approximately 1 × 10^7^ to 1 × 10^9^ particles/mL), and measurements were conducted using a ZetaView PMX 110 (Particle Metrix, Meerbusch, Germany) alongside ZetaView software (version 8.05.16 SP3). For TEM imaging, LEV samples were deposited onto holey carbon film-coated grids (Quantifoil, Saale-Holzland, Germany) using an Talos L120C (FEI, Hillsboro, OR, USA). The samples were then stained with 2% uranyl acetate for 20 s and subsequently blotted.

### 2.4. Preparation of LEVs Samples for Protein Identification

To verify the protein composition of LEVs, the pellet sample was lysed by adding 8 M Urea (Sigma-Aldrich, St. Louis, MO, USA) in conjunction with a protease inhibitor (Roche, Basel, Switzerland). The lysis process was conducted under conditions of 10% amplitude with pulses of 0.5 s on and off. Protein quantification was performed utilizing a BCA assay (PierceTM BCA Protein Assay Kit; Thermo Fisher Scientific, Waltham, MA, USA).

The LEVs were combined with 50 μL of 100 mM Tris-HCl (pH 8.0; Invitrogen, Thermo Fisher Scientific, Carlsbad, CA, USA) and 60 μg of the extract, followed by the addition of 61.8 μL of 8 M Urea (GE Healthcare, Chicago, IL, USA), which was then mixed thoroughly. For the reduction step, 2.02 μL of 1 M DTT (Sigma-Aldrich, St. Louis, MO, USA) was added to reach a final concentration of 10 mM, after which mixture was incubated at 56 °C in an incubator (Coretech, Seoul, Republic of Korea) for 30 min. To alkylate, 5.18 μL of 1 M IAA (Sigma-Aldrich, St. Louis, MO, USA) was added to achieve a final concentration of 25 mM, and the reaction was maintained at room temperature in the dark for 30 min. After this reaction, 1 mL of 100 mM Tris-HCl (pH 8.0; Invitrogen, Thermo Fisher Scientific, Carlsbad, CA, USA) was added to reduce the urea concentration to below 1 M, with the pH of the solution confirmed to be 8.0. For protein digestion, 12 μL of trypsin/Lys-C mix (Promega, Madison, WI, USA) at a concentration of 0.1 μg/μL was added and thoroughly mixed. The mixture was then incubated at 37 °C for 16 h. The reaction was terminated by the addition of 10 μL of 100% formic acid (Sigma-Aldrich, St. Louis, MO, USA), followed by mixing. The resulting sample was purified via solid-phase extraction (OASIS SPE Cartridge; Waters, Milford, CT, USA), subsequently dried, and prepared for further analysis.

### 2.5. LC–MS/MS Conditions for Proteomics Analysis

The dried sample was dissolved in 0.1% formic acid in water (Sigma-Aldrich, St. Louis, MO, USA) to a concentration of 1 μg/μL. Utilizing a Nano LC system (EASY-nLC 1200, Thermo Fisher Scientific, Waltham, MA, USA), an injection of 1 μL (equivalent to 1 μg) of the sample was performed. The sample was subsequently separated via an analytical column (EASY-Spray PepMap RSLC C18, 50 cm × 75 μm, 2 μm particle size; Thermo Fisher Scientific, Waltham, MA, USA) employing a gradual gradient of Solvent B (0.1% formic acid in acetonitrile) ranging from 5% to 95% over a period of 70 min. The flow rate condition was set up as 300 mL/min, with a total analysis duration of 70 min. Each sample was subjected to three replicate analyses. Detailed conditions for analysis are provided in Table 1A,B.

### 2.6. Protein Identification via Database Search

Protein identification was carried out using Proteome Discoverer (version 2.4; Thermo Fisher Scientific, MA, USA) with searches conducted against the *Leuconostoc mesenteroides* protein database. The database search parameters, including enzyme specificity, precursor and fragment mass tolerances, and fixed and variable modifications, are provided in Table 2.

### 2.7. Cytotoxicity Assessment of LEVs by WST-1 Assay

To investigate the impact of LEVs on cell viability, a cell proliferation reagent, the WST-1 assay (Water-Soluble Tetrazolium Salt), was employed. Human keratinocyte cells (HaCaT) were seeded to each well in 96-well plates with density of 1 × 10^4^ cells and then stabilized and cultured at 37 °C with 5% CO_2_ for 24 h. Subsequently, LEVs were diluted in culture medium to various concentrations, and 100 μL of each dilution was added to the respective wells. The wells were then maintained at 37 °C with 5% CO_2_ for 24 h. The WST-1 assay was conducted utilizing a cell viability assay kit (EZ-Cytox, DoGenBio Co., Ltd., Seoul, Republic of Korea) [35]. WST-1 reagent was diluted 1/10 in DMEM (Dulbecco’s Modified Eagle’s Medium, Welgene, Gyeongsan, Republic of Korea) supplemented with 1% penicillin/streptomycin (Gibco, Waltham, MA, USA). After removing the medium from each well, 100 μL of the reagent was added, and the plates were incubated at 37 °C with a 5% CO_2_ for 1 h. Absorbance at 450 nm was measured utilizing a microplate reader (Synergy HTX, BioTek, Winooski, VT, USA), and cell viability was subsequently calculated.

### 2.8. Analysis of LEVs on Skin Barrier Regulation via Real-Time PCR and ELISA

To analyze the effects of LEVs on skin barrier function and moisturizing properties, HaCaT were utilized. HaCaT cells were cultured and maintained in DMEM containing 10% FBS (Fetal Bovine Serum, Welgene, Gyeongsan, Republic of Korea) and 1% penicillin/streptomycin at 37 °C with a 5% CO_2_ environment. The cells were plated in 6-well dishes at a density of 5.0 × 10^5^ cells per each well and allowed to stabilize and culture for 24 h. Subsequently, the medium was replaced with serum-free medium to induce nutrient deprivation, and the cells were additionally maintained for 24 h. The experiment was performed under two conditions: without stimulation and with UV stimulation. In the non-stimulation condition, LEVs were diluted in serum-free medium to various concentrations and applied to starved cells, which were then cultured for an additional 24 h. The medium was subsequently collected, and the cells were washed with DPBS (Dulbecco’s Phosphate Buffered Saline; Welgene, Gyeongsan, Republic of Korea) and stored at −80 °C until further analysis. In the UV stimulation condition, starved cells were exposed to 20 mJ of UV radiation, followed by the addition of LEVs diluted at various concentrations in serum-free medium. The cells were then cultured for an additional 6 h, washed with DPBS, and stored at −80 °C until analysis.

### 2.9. Total RNA Extraction and mRNA Expression Analysis with Real-Time PCR

RNA was extracted from the obtained cells using the TaKaRa MiniBEST Universal RNA Extraction Kit (Takara, Kusatsu, Japan), and the RNA was quantified using a Nanodrop (KLAB, Daejeon, Republic of Korea). The quantified RNA was subjected to reverse transcription for complementary DNA (cDNA) synthesis using the High-Capacity RNA-to-cDNA Kit (Applied Biosystems, Thermo Fisher Scientific Inc., Waltham, MA, USA). Subsequently, cDNA synthesis and real-time polymerase chain reaction (PCR) were conducted employing the StepOnePlus Real-Time PCR System (Applied Biosystems, Foster City, CA, USA). For the analysis of Hyaluronan Synthase 3 (HAS3), FLG, and INV, TaqMan probes (Life Technologies, Waltham, MA, USA) and SYBR Green dye (BioRad, Hercules, CA, USA) were utilized.

### 2.10. Enzyme-Linked Immunosorbent Assay

The concentrations of Hyaluronic Acid (HA) were measured using ELISA kits (Hyaluronan DuoSet ELISA, R&D Systems, Minneapolis, MN, USA) following the manufacturer’s provided protocol. Briefly, 96-well microplates were coated overnight at room temperature with the capture antibody diluted in PBS. After washing, the plates were blocked through treatment with 1% BSA in PBS for 1 h. Standards and culture supernatant samples were subsequently placed into the wells and incubated at room temperature for 2 h. Following extensive washing, the detection antibody was applied for 2 h, and subsequently, streptavidin–HRP conjugate was added for 20 min. The colorimetric reaction was developed by adding substrate solution, and the reaction was stopped with 1N H_2_SO_4_. Absorbance was read at 450 nm with wavelength correction at 570 nm using a microplate reader (Synergy HTX, BioTek, Winooski, VT, USA). Hyaluronan concentrations were calculated from the standard curve generated using the supplied HA standards.

### 2.11. 3D Culture Model for Skin Barrier Improvement by LEVs

To assess whether LEVs genuinely enhance the skin barrier, alterations in barrier-related factors within the skin were quantified utilizing 3D cultured cells (Neoderm^®^, Tegoscience, Seoul, Republic of Korea). The positive control and test substances were diluted in medium and applied at a volume of 3.5 mL each to the culture layer, followed by incubation at 37 °C with 10% CO_2_ for 24 h. Post-incubation, the 3D cultured cells were processed to produce frozen blocks from which tissue sections were prepared. Immunofluorescence staining was conducted for FLG and INV (Table 3). Each experiment was performed in duplicate, with the negative control using medium and the positive control consisting of 200 μg/mL of L-ascorbic acid.

### 2.12. Immunofluorescence Staining

The frozen block was sectioned at a thickness of 5 μm using a −20 °C cryostat to obtain sections. The sections were fixed in an Acetone:MeOH (1:1) fixative at −20 °C and washed three times with 1X PBS. The washed sections were blocked with 1% BSA (Bovine Serum Albumin; Sigma-Aldrich, St. Louis, MO, USA) and subsequently incubated with the primary antibody overnight at 4 °C. The secondary antibody was applied at room temperature for 1 h, followed by mounting with fluorescence mounting medium [36]. The samples were then observed and analyzed under a 200X microscope.

### 2.13. Statistical Analysis

Statistical analysis was conducted utilizing the IBM SPSS software (version 25, IBM, Armonk, NY, USA). For comparisons between two groups, an initial normality assessment was performed; if the data adhered to a normal distribution, an independent samples *t*-test was subsequently applied. For analyses involving three or more groups, either one-way analysis of variance (ANOVA) was employed. In cases where significant differences were observed, Tukey’s multiple comparison post hoc test was conducted. Results are presented as mean ± standard deviation (SD), with substantial differences denoted by asterisks (*, **, or ***). The threshold for statistical significance was established at *p* < 0.05.

## 3. Results

### 3.1. EVs Secretion by Leuconostoc mesenteroides

**Figure 1:**
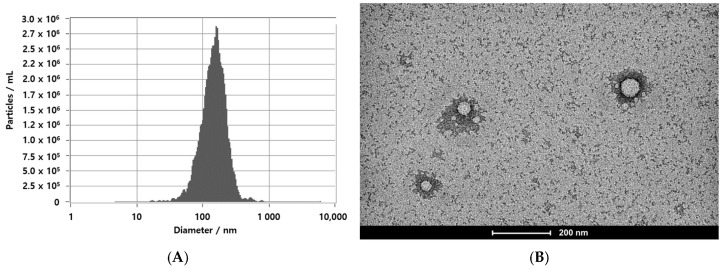
Characterization of LEVs. (**A**) Size distribution of LEVs measured by Nanoparticle Tracking Analysis (NTA). (**B**) Morphology of LEVs observed by Transmission Electron Microscopy (TEM). Scale bar: 200 μm.

EVs released from *Lactiplantibacillus* sp. have been commonly reported to fall within the nanoscale range, typically between 20 and 200 nm [37]. In the present study, Nanoparticle Tracking Analysis (NTA) was employed to assess the size and concentration of LEVs (Figure 1A). The concentration of LEVs, which reside within the particle size range of approximately 50–200 nm, was verified to be 1.8 × 10^10^ particles/mL. Transmission Electron Microscopy (TEM) further confirmed the size and morphology of the samples obtained via the TFF system (Figure 1B).

### 3.2. Proteomic Analysis of LEVs

**Figure 2:**
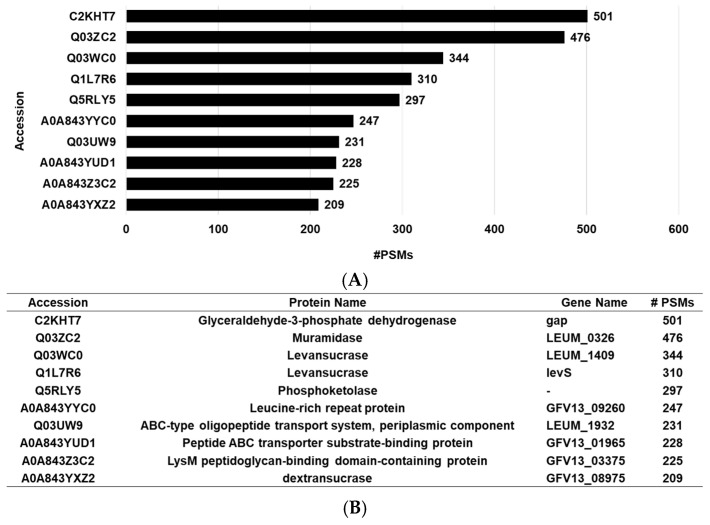
Proteomic analysis of LEVs. (**A**) Relative abundance of proteins within LEVs, determined by the number of peptide-spectrum matches (PSMs) for each protein divided by the total PSMs detected in the sample. (**B**) Relative abundance profiles of the top 10 identified proteins. All proteomic analyses were performed in triplicate (*n* = 3).

Proteomic analysis through the LC-MS/MS instrument has been successfully applied to identify the protein composition of EVs derived from *Lactobacillus* sp., highlighting its usefulness in characterizing EV constituents and clarifying their biological relevance [38]. To determine the protein composition within EVs and elucidate their biological functions and potential efficacy, an analysis was conducted utilizing LC-MS/MS (Figure 2A). Consequently, a total of 569 proteins were examined, among which C2KHT7 (Glyceraldehyde-3-phosphate dehydrogenase, GAPDH), Q03ZC2 (Muramidase), and Q03WC0 (Levansucrase) were identified as the three predominant proteins (Figure 2B).

### 3.3. Gene Ontology (GO) Analysis of Proteins in LEVs

**Figure 3:**
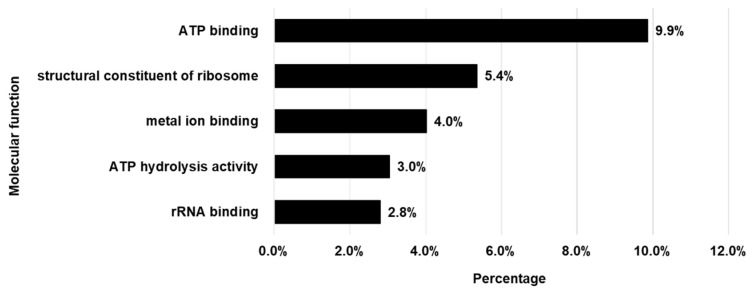
GO molecular function analysis of proteins within LEVs. The graph illustrates the five primary molecular functions of the proteins as classified by GO. The percentages indicate the proportion of proteins attributed to each function in relation to the total number of proteins identified.

GO analysis has been recognized as a practical approach for interpreting the functional characteristics of proteins identified in lactic acid bacteria-derived EVs, thereby providing insights into their biological relevance [39]. By confirming the functional characteristics of the identified proteins, an analysis was conducted to interpret how EVs might be associated with specific efficacies. As a result of performing GO analysis, the top three primary functions in the Molecular Function category were ATP binding, structural constituent of ribosome, and metal ion binding (Figure 3).

### 3.4. Cell Viability

**Figure 4:**
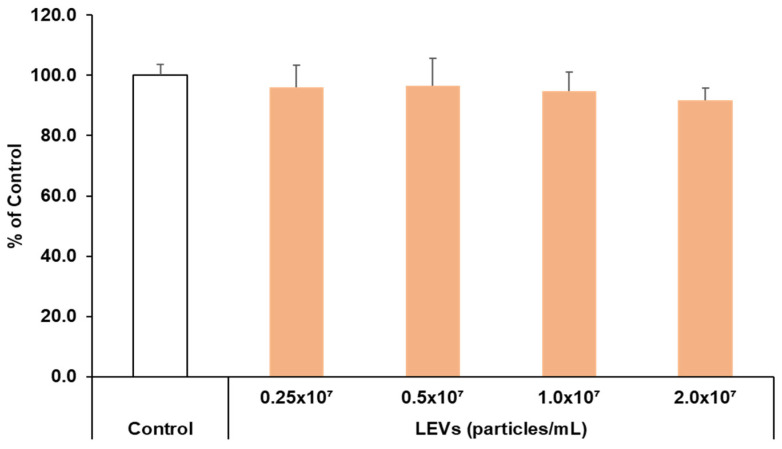
Cytotoxicity evaluation of LEVs in HaCaT cells. HaCaT cells were subjected to different concentrations of LEVs (0.25 × 10^7^ to 2.0 × 10^7^ particles/mL) for a duration of 24 h. Cell viability was measured by the WST-1 assay. Results are expressed as the mean ± standard deviation (SD) derived from three independent experiments and are expressed as a percentage relative to the control. Statistical analysis was performed using SPSS software; significance levels were analyzed by ANOVA with Tukey’s post hoc test.

To confirm the safety of LEVs, a cytotoxicity assessment was conducted. LEVs were diluted to various concentrations and applied to HaCaT keratinocytes. The results indicate that cell viability exceeded 90% even when treated with LEVs at concentrations up to 2.0 × 10^7^ particles/mL (Figure 4). In general, when the cell viability exceeded 80% after sample treatment, it can be determined that cell cytotoxicity did not occur. Therefore, it can be regarded that there was no cytotoxicity at all concentrations of LEVs treated. Accordingly, subsequent experiments were performed using LEVs concentrations of 2.0 × 10^7^ particles/mL or less.

### 3.5. Skin Barrier Moisturizing Factor Analysis

**Figure 5:**
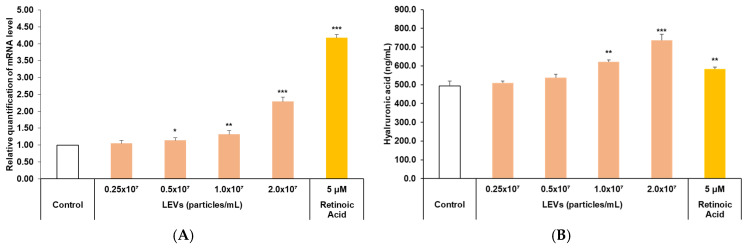
HAS3 expression and hyaluronic acid production in HaCaT cells. HaCaT cells received treatment with LEVs at concentrations ranging from 0.25 × 10^7^ to 2.0 × 10^7^ particles/mL for 24 h under serum-free conditions. 5 μM retinoic acid was employed as a positive control. (**A**) The mRNA expression level of HAS3 was quantified through real-time PCR. (**B**) The levels of secreted hyaluronic acid in the culture medium were measured using an ELISA assay. Results are expressed as the mean ± standard deviation (SD) from three independent experiments. Statistical evaluations were conducted using SPSS; statistical differences were analyzed by ANOVA with Tukey’s post hoc test (* *p* < 0.05, ** *p* < 0.01, *** *p* < 0.001 versus Control).

Hyaluronic acid is recognized for its critical role in moisture retention within the skin, and as such, it is frequently examined as a factor contributing to skin hydration. Consequently, the expression level of HAS3 gene, an enzyme responsible for the synthesis of hyaluronic acid, was evaluated. Additionally, hyaluronic acid levels in the cell culture supernatant were measured using ELISA to assess the skin moisturizing efficacy of LEVs (Figure 5). Regarding the expression level of the HAS3 gene, it was observed that at the highest LEVs treatment concentration of 2.0 × 10^7^ particles/mL, there was a notable increase of approximately 2.3-fold compared to the control group (Figure 5A). In the quantitative analysis of hyaluronic acid within the culture supernatant, a significant increase of approximately 1.5-fold was observed at the same LEVs concentration of 2.0 × 10^7^ particles/mL compared to the control (Figure 5B). Based upon these results, LEVs are considered to have a potential role in enhancing skin hydration.

### 3.6. Improvement of Skin Barrier Function

**Figure 6:**
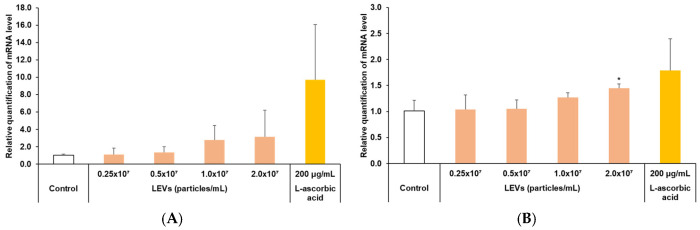
Involucrin and filaggrin expression in HaCaT cells. (**A**) Involucrin and (**B**) Filaggrin mRNA expression levels were quantified by real-time PCR after treatment with LEVs (ranging from 0.25 × 10^7^ to 2.0 × 10^7^ particles/mL) under serum-free conditions. 200 μg/mL L-ascorbic acid served as the positive control. Results are expressed as the mean ± standard deviation (SD) from three independent experiments. Statistical analyses were conducted using SPSS; statistical differences were analyzed by ANOVA with Tukey’s post hoc test (* *p* < 0.05 versus Control).

Among the various factors involved in skin barrier formation, INV is recognized to play a significant role in establishing keratinocyte membranes, and FLG is acknowledged for its contribution to maintaining the skin barrier through binding to keratin fibers within epithelial cells. Consequently, the expression levels of the INV and FLG genes were assessed to ascertain whether LEVs could enhance skin barrier function (Figure 6). In the case of INV gene expression, at the highest LEV treatment concentration of 2.0 × 10^7^ particles/mL, an approximate 3.13-fold increase was observed compared to the control group. Although this increase was proportional to the LEV concentration and the rate of growth was higher, the standard deviations across each test group were considerable, resulting in the difference not reaching statistical significance (Figure 6A). Regarding FLG gene expression, at the maximum LEV treatment concentration of 2.0 × 10^7^ particles/mL, a notable increase of approximately 1.45 times was detected relative to the control group (Figure 6B).

### 3.7. Expression of Skin Barrier Factors in a 3D Skin Model

**Figure 7:**
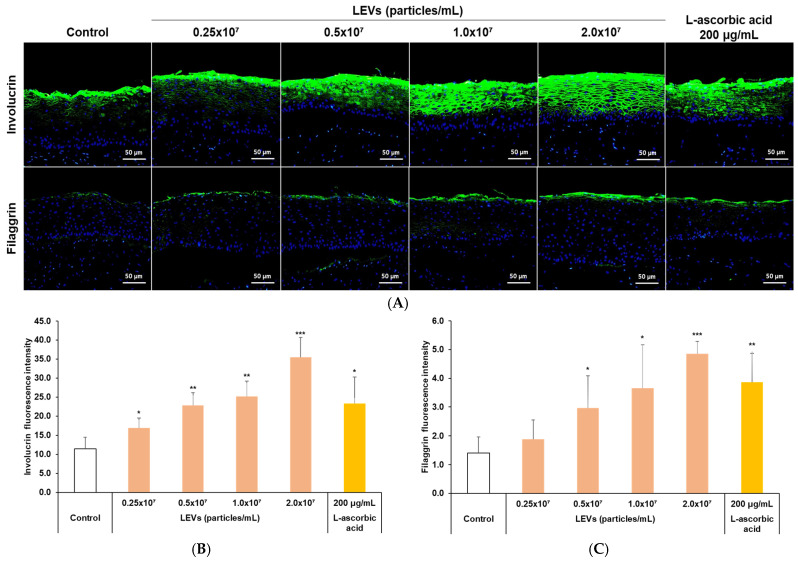
Assessment of skin barrier protein expression by LEVs in a 3D cultured full-thickness skin model. (**A**) Representative immunofluorescence images illustrating involucrin and filaggrin expression following 24 h of treatment with LEVs (ranging from 0.25 × 10^7^ to 2.0 × 10^7^ particles/mL) or L-ascorbic acid (200 μg/mL). (**B**,**C**) Graphs displaying the quantified fluorescence intensity of (**B**) involucrin and (**C**) filaggrin. Sections were prepared from frozen tissue blocks, stained with primary antibodies and FITC-conjugated secondary antibodies, and counterstained with DAPI. Images were acquired at 200X magnification. Results are expressed as mean ± SD (*n* = 3). Statistical analysis was performed using SPSS; * *p* < 0.05, ** *p* < 0.01, *** *p* < 0.001 vs. Control. Scale bar: 50 μm.

To evaluate the effects of LEVs on skin barrier enhancement, a three-dimensional cell culture system was applied. The 3D skin model was treated with four different concentrations of LEVs. Subsequently, 3D cultured skin cross-sections were prepared, and fluorescence staining was performed to visualize skin barrier factor components (Figure 7A). Fluorescence intensities of stained skin barrier factor were compared to evaluate changes in their expression levels induced by LEVs treatment. The experimental results revealed that, relative to the negative control, the expression levels of INV (Figure 7B) and FLG (Figure 7C) increased in a dose-dependent manner in response to LEVs treatment.

### 3.8. Protection of Skin Barrier Function

**Figure 8:**
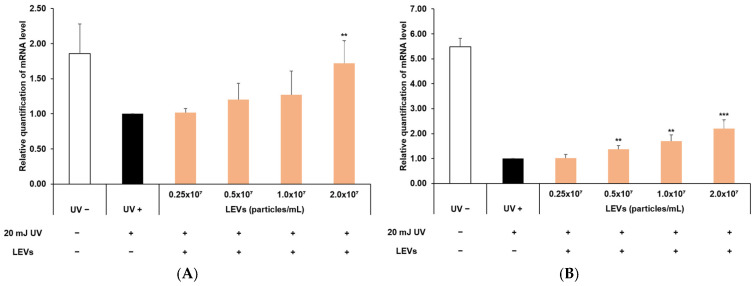
Involucrin and filaggrin expression in UV-irradiated HaCaT cells. (**A**) Involucrin and (**B**) Filaggrin mRNA expression levels were measured by real-time PCR after treatment with LEVs (0.25 × 10^7^ to 2.0 × 10^7^ particles/mL) under UVB-irradiated conditions. Non-irradiated (UV−) and UV-irradiated (UV+) cells were used as controls. Results are expressed as the mean ± standard deviation (SD) based on three independent experiments. Statistical analysis was performed using SPSS; significance was analyzed by ANOVA with Tukey’s post hoc test (** *p* < 0.01, *** *p* < 0.001 versus UV+ control).

Furthermore, an experiment was conducted to assess whether LEVs contribute to protecting the skin barrier in instances of damage caused by UV radiation (Figure 8). Keratinocytes were exposed to 20 mJ of UVB, and the expression levels of the INV and FLG genes were quantified with and without LEV treatment. At the highest LEV concentration of 2.0 × 10^7^ particles/mL, a significant increase of approximately 1.72-fold for the INV gene compared to the control was observed (Figure 8A). For the FLG gene, a substantial increase of approximately 2.2-fold was noted at the same concentration (Figure 8B).

## 4. Discussion

Recent studies indicate that exosomes are increasingly recognized as promising therapeutics for skin tissue regeneration, achieved through the regulation of inflammation, enhancement of epithelial reformation, and restoration of the skin barrier [40,41]. In studies on microorganisms, EVs from *Lactobacillus plantarum* were shown to reduce inflammatory cytokine expression and to increase skin barrier-related factors such as FLG and LOR [42]. Furthermore, it has been reported that these EVs can reduce oxidative stress in cells damaged by UV radiation and promote collagen synthesis, thereby contributing to anti-aging effects [43].

Microorganisms derived from the *Camellia japonica* flower, known for its beneficial effects on skin, were used to isolate LEVs, which were subsequently evaluated for their effects on the skin barrier. LEVs were purified by tangential flow filtration (TFF) and subsequently characterized for particle concentration, size, and morphology using TEM and NTA. Additionally, we performed a proteomic study of LEVs and determined proteins including GAPDH, Muramidase, and Levansucrase were present at relatively high abundance. GAPDH is a well-known multifunctional enzyme involved in energy metabolism and the regulation of cellular stability [44]. The role of Muramidase as an innate antimicrobial factor has been described, with evidence indication its contribution to microbial balance on the skin and to barrier homeostasis [45], and Levansucrase is associated with exopolysaccharide (EPS) biosynthesis [46], and has been shown to support moisture retention, promote hydration, and modulate mucosal immune responses [47,48]. The proteins profile identified through the proteomics analysis offers foundational insight into the molecular traits that LEVs from DB-21 may possess, and this information can serve as contextual background when interpreting the hydration- and barrier-related outcomes observed in this study. However, it is more appropriate to view these proteins as related to the biological environment and the molecular characteristics that LEVs may exhibit, rather than as directly contributing to functional changes.

According to the GO analysis, major functions were ATP binding effect, structural constituent of ribosome, and metal ion binding effect. ATP-binding proteins serve key roles in both energy production and in maintaining cellular communication and stability [49], which is consistent with the multifunction of GAPDH. The structural proteins of ribosomes are central to protein synthesis and participate markedly in promoting cell division and tissue regeneration. Some of these proteins have been observed to contribute to preventing collagen degradation through facilitating DNA repair after UV-induced damage, and reducing MMP-1 expression [50]. Metal ion-binding proteins, through their interaction such as zinc, magnesium, and copper ions are engaged in biological pathways related to differentiation migration, collagen assembly and the regulation of inflammatory responses [51]. These functional categories identified through GO analysis provide a broad understanding of the potential molecular characteristics of LEVs and are consistent with previous reports suggesting that microbe-derived EV proteins can be involved in cell differentiation, modulation of inflammatory signals, and tissue regeneration. These molecular properties are confirmed by previous studies showing that stem cell-derived exosomes promote skin tissue regeneration and barrier protection, and that EVs derived from *Lactobacillus* sp. contribute to maintaining skin microbial balance and barrier improvement through anti-inflammatory and antioxidant effects [48,49]. These EV-related studies also support the possibility that LEVs may positively affect skin moisture retention and barrier strengthening. Therefore, based on the GO analysis results of LEVs, we determined at the molecular level through in vitro and 3D skin model studies that LEVs have a positive effect on moisture and barrier enhancement.

First of all, we examined the expression level of HAS3, an enzyme involved in hyaluronic acid production and regarded as a key factor in maintaining skin moisture. Consequently, cells treated with LEVs showed a significant upregulation of HAS3 expression, accompanied by an evident rise in hyaluronic acid levels relative to the control group. These results suggest that LEVs regulate HAS3 expression at the transcriptional stage and concurrently stimulate hyaluronic acid synthesis, which contributes to enhancing the skin’s moisture-retaining ability. Afterward we confirmed that the expression levels of FLG and INV, which are involved in skin barrier formation, increased after LEV treatment. Furthermore, in the three-dimensional skin equivalent model, their expression increased proportionally with the LEV concentration, indicating that LEVs promote barrier-related protein expression and strengthen the skin barrier, thereby contributing to overall skin protection. In addition to improving the barrier, another important aspect of skin protection involves defense against ultraviolet (UV) radiation. Among various environmental stressors, UV is known to exert harmful effects on skin barrier maintenance [52]. When LEVs were applied, the expression of FLG and INV was restored, and this recovery appears to support the maintenance of skin barrier resilience under UV-induced stress.

In conclusion, extracellular vesicles derived from *Leuconostoc mesenteroides* DB-21, isolated from *Camellia japonica*, increased the expression levels of molecular markers associated with skin moisturization and barrier function, and supported the protection of these molecular markers against UV-induced damage. Through this study, we presented the initial correlation between LEVs proteins and functional responses. In the future, elucidating the mechanisms by which proteins within LEVs contribute to biological changes, and, if direct efficacy is confirmed through in vivo and clinical research, LEVs are expected to have industrial potential as promising microbiome-based materials for skin moisturization and barrier improvement.

## Figures and Tables

**Table 1:** LC-MS/MS analysis conditions for LEVs. (**A**) Nano liquid chromatography conditions, (**B**) Mass spectrometry analysis parameters for LEVs using Q Exactive Plus.

(A)			
Parameter	Setting		
Analytical Column	EASY-Spray PepMap RSLC C18 (50 cm × 75 μm, 2 μm; Thermo Fisher Scientific, Waltham, MA, USA)		
Mobile phase	A: 0.1% Formic acid in water B: 0.1% Formic acid in Acetonitrile		
Flow rate	300 mL/min		
Injection Volume	1 μL (1 μg peptide)		
Column Temperature	50.0 °C		
Autosampler Temperature	4.0 °C		
Elution condition (Gradient)	Time (min)	A (%)	B (%)
	0	95	5
	45	50	50
	48	5	95
	52	5	95
	55	95	5
	70	95	5
(**B**)			
Parameter	Setting		
Equipment	Q Exactive Plus Hybrid Quadrupole-Orbitrap Mass Spectrometer (Thermo Fisher Scientific, USA)		
Ion Mode	ESI		
Full MS condition			
Resolution	70,000		
Scan Range	350–2000 *m*/*z*		
Maximum IT	120 ms		
Polarity Volume	Positive		
MS/MS condition (DDA: Data Dependent Acquisition)			
Resolution	17,500		
AGC	5.00 × 10^5^		
Isolation width	1.2 *m*/*z*		
Top N	20		
NCE	25%		
Maximum IT	80 ms		
Dynamic Exclusion	30 s		

**Table 2:** Search parameters for protein identification.

Parameter	Value
Precursor mass tolerance	10 ppm
Fragment mass tolerance	0.02 Da
Enzyme	Trypsin
Miss cleavage	2
Static modification	Carbamidomethyl (C)

**Table 3:** Antibody information.

1st Antibody		2nd Antibody
Marker	Host	
Filaggrin	Rabbit	FITC–anti rabbit
Involucrin	Rabbit	FITC–anti rabbit

## Data Availability

The datasets generated and analyzed during the current study are proprietary to the company and are not publicly available due to commercial confidentiality. All data necessary to interpret and evaluate the findings of this study are included within the article.
